# Different Chronic Stress Paradigms Converge on Endogenous TDP43 Cleavage and Aggregation

**DOI:** 10.1007/s12035-023-03455-z

**Published:** 2023-07-14

**Authors:** Niccolò Candelise, Daniela Caissutti, Henri Zenuni, Valentina Nesci, Silvia Scaricamazza, Illari Salvatori, Zaira Spinello, Vincenzo Mattei, Tina Garofalo, Alberto Ferri, Cristiana Valle, Roberta Misasi

**Affiliations:** 1 Department of Experimental Medicine, University La Sapienza, 00185 Rome, Italy; 2 IRCCS Fondazione Santa Lucia, 00179 Rome, Italy; 3 Department of Systems Medicine, Tor Vergata” University of Rome, 00133 Rome, Italy; 4 Biomedicine and Advanced Technologies Rieti Center, Sabina Universitas, 02100 Rieti, Italy; 5 Institute of Translational Pharmacology (IFT), Consiglio Nazionale Delle Ricerche (CNR), 00185 Rome, Italy

**Keywords:** TDP43, Chronic stress, Amyotrophic Lateral Sclerosis, Prion, Thioflavin

## Abstract

The TAR-DNA binding protein (TDP43) is a nuclear protein whose cytoplasmic inclusions are hallmarks of Amyotrophic Lateral Sclerosis (ALS). Acute stress in cells causes TDP43 mobilization to the cytoplasm and its aggregation through different routes. Although acute stress elicits a strong phenotype, is far from recapitulating the years-long aggregation process. We applied different chronic stress protocols and described TDP43 aggregation in a human neuroblastoma cell line by combining solubility assays, thioflavin-based microscopy and flow cytometry. This approach allowed us to detect, for the first time to our knowledge in vitro, the formation of 25 kDa C-terminal fragment of TDP43, a pathogenic hallmark of ALS. Our results indicate that chronic stress, compared to the more common acute stress paradigm, better recapitulates the cell biology of TDP43 proteinopathies. Moreover, we optimized a protocol for the detection of *bona fide* prions in living cells, suggesting that TDP43 may form amyloids as a stress response.

**Supplementary Information:**

The online version contains supplementary material available at 10.1007/s12035-023-03455-z.

## Introduction

Inclusions of protein aggregates containing the TAR-DNA binding protein-43 (TDP43) are pathogenic hallmarks in the vast majority of sporadic cases of Amyotrophic Lateral Sclerosis (ALS) and in a substantial proportion of Frontotemporal Lobar Degeneration (FTLD) [1–4]. TDP43 proteinaceous inclusions are formed after its misfolding, mislocation and subsequent aggregation [5, 6]. Whereas in physiological conditions TDP43 resides mainly in the nucleus, it was shown to be mobilized in the cytosol upon stress induction both in cellular and animal models of ALS [2, 5, 6]. Aggregated cytosolic TDP43 is phosphorylated, ubiquitinated and cleaved in C-terminal fragments of 35 kDa, 25 kDa (termed CTF-35, CTF-25 respectively) and lower molecular weights [7–14]. Most pathology-associated mutations are found in the aggregating prone, prion-like C-terminal domain, which overlaps with TDP43 cleaved products [15, 16].

A growing body of evidence is indicating protein misfolding and aggregation in a prion-like fashion as the main toxic mechanism responsible for the onset of most neurodegenerative diseases [3, 4, 17]. The prion-like aggregation pathway consists of a misfolding event that leads to the formation of a β-sheet enriched, active seed. This misfolded protein, in turn, forces the conversion of its native counterpart into β-enriched structures, forming cross-β bondings that eventually coalesce into the characteristic amyloid fibrils found in post-mortem brains of patients affected by neurodegenerative diseases. Notably, in ALS and in other similar pathologies, protein aggregation appears to be an early event occurring during the pre-symptomatic stages of the disease [6, 18–20]. Amyloid-like depositions of TDP43, positive to the cross-β binding dye Thioflavin-S (ThS), were found in the brain of a subset of ALS patients [4], suggesting a prion-like aggregation mechanism for TDP43 toxicity. Thioflavins are benzothiazole molecules that exhibit a shift in excitation and emission maxima (from 385 to 450 nm and from 485 nm to 482, respectively) when bound to the characteristic cross-ꞵ structure that defines amyloid fibrils [4, 9].

In cellular models of ALS, acute stress paradigms involving osmotic, oxidative and metabolic alterations were shown to cause TDP43 translocation in the cytoplasm [21–24], where it associates with stress granules or perform liquid–liquid phase separation [2, 6, 25–29]. To date, cell-based studies aimed to dissect TDP43 behaviour were conducted following acute insults. Albeit this experimental paradigm elicits a robust biological response in terms of TDP43 localization and aggregation, is far from representing the slow and persistent alterations occurring in the years-long disease process, which are better mirrored by long-term, chronic stress conditions [30, 31].

The aim of this work was to analyze endogenous TDP43 mobilization, cleavage and aggregation state upon acute and chronic insults in order to evaluate which stress paradigms better recapitulate the course of the disease. To this end, we applied a panel of acute and chronic stressors to human neuroblastoma SH-SY5Y cells. We opted to perform our experiment on a neuronal cell line without any genetic manipulation commonly used in literature for TDP43 biology (e.g., plasmid transfection [7, 8]) nor any differentiation protocol (e.g., induced pluripotent stem cells [30] or retinoic acid-induced differentiation [25]) to keep the system as close as possible to the cellular physiological state. We optimized a fast and reliable flow cytometry assay to detect *bona fide* cross-β structures in living cells by staining with the amyloid-specific dye ThS and demonstrate a differential effect of acute versus chronic stress in terms of TDP43 solubility and cleavage products, along with a difference in the overall amyloid burden. Our results point toward a physiological production of TDP43 cleaved fragments, along with an increase in amyloid structures only upon chronic treatment, suggesting a putative physiological stress response based on TDP43 aggregation, typically referred as a pathogenic event. Furthermore, our results indicate that long-term chronic stress paradigms are better suited to study early events in cellular models of ALS and possibly in neurodegenerative disease models in general.

## Materials and Methods

### Cell Culture

Human neuroblastoma cell line SH-SY5Y was cultured at 37 °C, 5% CO_2_ in Dulbecco’s Modified Eagle Medium supplemented with nutrient mixture F-12 (DMEM/F-12, 11,320,033, Gibco, Thermo Fisher Scientific) containing L-Glutamine and 2,438 g/L Sodium Carbonate, supplemented with 10% Fetal Bovine Serum (FBS, 35–015-CV, Corning) and 5% of Penicillin/Streptomycin (ECB3001D, Euroclone). Unless otherwise stated, 3 to 5 × 10^5^ cells were plated in 6 well culture plates (3516, Corning), each well having a surface of 9.5 cm^2^. The day after, the medium was removed and cells were washed in 1 × Dulbecco’s Phosphate Buffer Solution (PBS, 14,040–091, Gibco, Thermo Fisher Scientific) and incubated in DMEM/F-12 with the addition of stressors for either acute or chronic treatment. The maximum cell passage used was 10.

### Stress Induction and Cytotoxicity Assay

Concentrations and time of exposure for acute stress were based on previously published results on SH-SY5Y cells when available [32–35]. Acute treatments were performed following incubation with 0.5 mM, 1 mM and 2 mM Sodium Arsenite (Ars, S7400, Sigma) for 1 h, or 0.6 M, 1 M and 1.2 M D-Sorbitol (Sorb, S1876, Sigma) for 2 h**,** or 2 mM and 2.5 mM *N*,*N*′-dimethyl-4,4′-bipyridinium dichloride (Paraquat, PQ, 856,177, Sigma) overnight (approximately 16 h) and compared to untreated SH-SY5Y cells. Each compound was suspended in water and diluted in cell culture medium to the final concentration. For chronic treatments, the time of exposure was set at 72 h and the concentrations of stressors were as follows: Ars 10 µM, 15 µM, 20 µM; Sorb 80 mM, 100 mM, 120 mM; PQ 0.1 mM, 0.15 mM, 0.2 mM. Additionally, Serum deprivation (SD), achieved by reducing FBS to 1% or 0.1%, was further tested as a source of chronic environmental stress [32, 36].

Cell viability was assessed by 3-(4,5-dimethylthiazol-2-yl)-5-(3-carboxymethoxyphenyl)-2-(4-sulfophenyl)-2H-tetrazolium (MTS) assay (CellTiter 96® AQ_ueous_ One Solution Cell Proliferation Assay, G3580, Promega). 20 × 10^3^ cells were plated in a 96-well plate (3599, Costar) and treated with growing concentrations of acute and chronic treatments in culture medium (Suppl. Figure 1).

Cells were plated in quadruplicates and allowed to grow at the indicated time of exposure and concentration at 37 °C, 5% CO_2_. After treatments, 20 µL of MTS were added and absorbance measurements at 490 nm were acquired after 90 min of incubation at 37 °C through a multilabel counter Victor 3 V plate reader (1420, Perkin Elmer). Since the MTS assay reflects the metabolic activity of the cells, concentrations that provided the best balance between cell death and viability were selected as working concentrations and listed in Table 1.

**Table 1:** Acute and chronic stress paradigms. The table indicates the effect. the concentration and the time of exposure of different stressors administered in acute and chronic conditions. References report recent usage of each stressor to human SH-SY5Y neuroblastoma cells

Stressor	Effect	Acute Treatment	Chronic Treatment	References
Serum Deprivation (SD)	Lack of growth factors; Reduced nutrition	/	1%. 72 h	[32]
Sodium Arsenite (Ars)	Oxidative stress; Mitochondrial disruption	0.5 mM 1 h	20 µM 72 h	[33]
D-Sorbitol (Sorb)	Hyperosmotic stress; Small molecules leakage	1.2 M 2 h	120 mM 72 h	[34]
Paraquat (PQ)	Oxydative stress; ROS production	2 mM O.N	0.2 mM 72 h	[35]

### Biochemical Fractionation and Western Blot

For Western blot, 3 × 10^5^ cells were plated for each condition. After treatment, cells were harvested by scraping in cell culture medium and harvested by centrifugation. The pellet was lysed in 100 µL of high salt RIPA buffer containing 50 mM Tris–HCl pH 8.5 mM EDTA pH 8, 250 mM NaCl, 1% Triton-X (T9284, Sigma), 0.1% SDS, 0.25% Na-Deoxycholate and supplemented with protease inhibitors cocktail (P8340, Sigma) and incubated for 30’ on ice. The lysate was centrifuged at 14,000 × g for 30’ at 4 °C to obtain a RIPA soluble fraction (Sol) and a RIPA insoluble fraction (Ins). Ins fraction was suspended in 100 µL of 1 × sample buffer containing 50 mM Tris–HCl, pH 6.8, 50 mM β-Mercaptoethanol, 10% Glycerol, 1% SDS and Bromophenol Blue, and boiled at 100 °C for 10’. 10 µg of proteins from the Sol fraction and an equivalent amount of Ins fraction were loaded onto a 12.5% SDS-PAGE gel. After electrophoresis, transfer, and blocking in 5% dry milk in TBS-T containing 20 mM Tris, 150 mM NaCl and 0.1% Tween 20 (P2287, Sigma), membranes were incubated overnight at 4 °C under mild agitation with either C-terminal polyclonal antibody (amino acids 203–209) anti-TDP43 (10,782–2-AP, Proteintech) or anti-GAPDH antibody (10,494–1-AP, proteintech). The next day, membranes were washed and incubated with anti-IgG Horseradish peroxidase-conjugated secondary, washed again and incubated for 5’ with Clarity Western ECL Substrate (170–5061, Bio-Rad). Images were acquired with the iBright CL1000 Imaging system (Invitrogen). Densitometry was performed on biological triplicates by ImageJ software.

### Flow Cytometry

To assess the overall amyloid burden of cells after acute and chronic stress, we coupled ThS (T1892, Sigma) fluorescence emission with flow cytometry. SH-SY5Y neuroblastoma cells were seeded in 6-well plates at a density of 5 × 10^5^ cells/well and subjected to the panel of stressors as mentioned above. At the end of the treatments, 0.01% ThS in 50% Ethanol was added in each well and incubated at 37 °C, 25% CO_2_ for 1 h. Next, both cells and supernatant were collected and washed twice in 1 × PBS before performing flow cytometric analysis through CytoFLEX flow cytometer (Beckman Coulter). To exclude cell debris, cells were first gated (P1) by Forward Scattering (FSC) and Side Scattering (SSC) signals (upper left); next, height versus area of FSC dot plot was used to exclude doublet events or cell clumps (P2, upper right); Cells in P2 were gated again (P3 and P4) by FSC-A and ThS emission fluorescence, measured on a FITC detector (lower left). ThS fluorescence intensity of both P3 and P4 cells vs count was represented in histogram plots (lower right).

### ThS Staining and Immunofluorescence

3 × 10^5^ SH-SY5Y cells were plated on a 10 mm poly-D-Lysine (Gibco, cat #A3890401) glass and incubated with adequate concentration for the reported time for both acute and chronic treatments. Cells were washed once in PBS, fixed in 4% Paraformaldehyde (Merck, cat #104,003), permeabilized in 0.1% Triton-X and blocked in 3% Bovine Serum Albumin (BSA, Sigma, cat #2153) and 0.01% Triton-X in PBS. Cells were next incubated still at 4 °C overnight with anti-TDP43 (10,782–2-AP, Proteintech). The next day, cells were washed in PBS and incubated still for 1 h in anti-rabbit IgG raised in donkey (Merck, cat #AP182C). For ThS staining, following washings after secondary antibody, cells were incubated still for 10’ in 0.025% ThS in 50% Ethanol, then rinse-washed in decreasing Ethanol concentrations (50%, 25% and 12.5%). Cells were washed again in PBS before the incubation in DAPI solution (Thermo Fisher Scientific, cat # 62,248). Images were acquired from biological triplicates for each condition at 100 × magnification using an Olympus BX53 fluorescent microscope with the diagnostic instrument camera model 2.3.1.

### Subcellular Fractionation

Nuclear and cytoplasmic fraction were extracted following an established protocol with minor modifications [37]. 5 × 10^6^ cells were plated for each condition. After stress induction, cells were incubated for 5’ on ice in a lysis buffer containing 50 mM KCl, 25 mM HEPES pH 7.8, 0.5% IGEPAL CA-630 (Sigma, cat #I-3021) and 125 µM dithiothreitol, 1 mM Na_3_VO_4_ and 1 × protease inhibitor cocktail (Sigma, cat #P8340). Lysates were centrifuged at 10,500 × g for 10’ at 4 °C and the supernatant was kept as the cytosolic fraction. The pellet was washed in a buffer containing 50 mM KCl, 25 mM HEPES pH 7.8, 125 µM DTT, 1 mM Na_3_VO_4_ and 1 × protease inhibitor cocktail and centrifuged at 10,500 × g for 10’ at 4 °C. Supernatant was removed, the pellet fraction containing nuclei was resuspended in 1 × sample buffer and sonicated in a bath sonicator for 90’’ at maximum amplitude before boiling for 10’. 15 µg of nuclear and cytoplasmic fractions were loaded onto a 10% polyacrylamide gel and probed for: TDP43, phosphoTDP43 (Ser409/410, proteintech, cat # 22,309–1-AP), the cytosolic marker Beta-Tubulin (Sigma, cat # T8328) and the nuclear marker SP-1 (PEP 2, Santa Cruz Biotechnology, cat # SC59).

### Statistical Analysis

Data were analyzed using GraphPad Prism 9 software. One-way ANOVA followed by the Brown-Forsythe test for variance homogeneity was performed. Multiple comparisons were performed using the Dunnett test against untreated control for the evaluation of statistical differences among samples.

## Results

### Acute and Chronic Stress Cytotoxicity

**Fig. 1:**
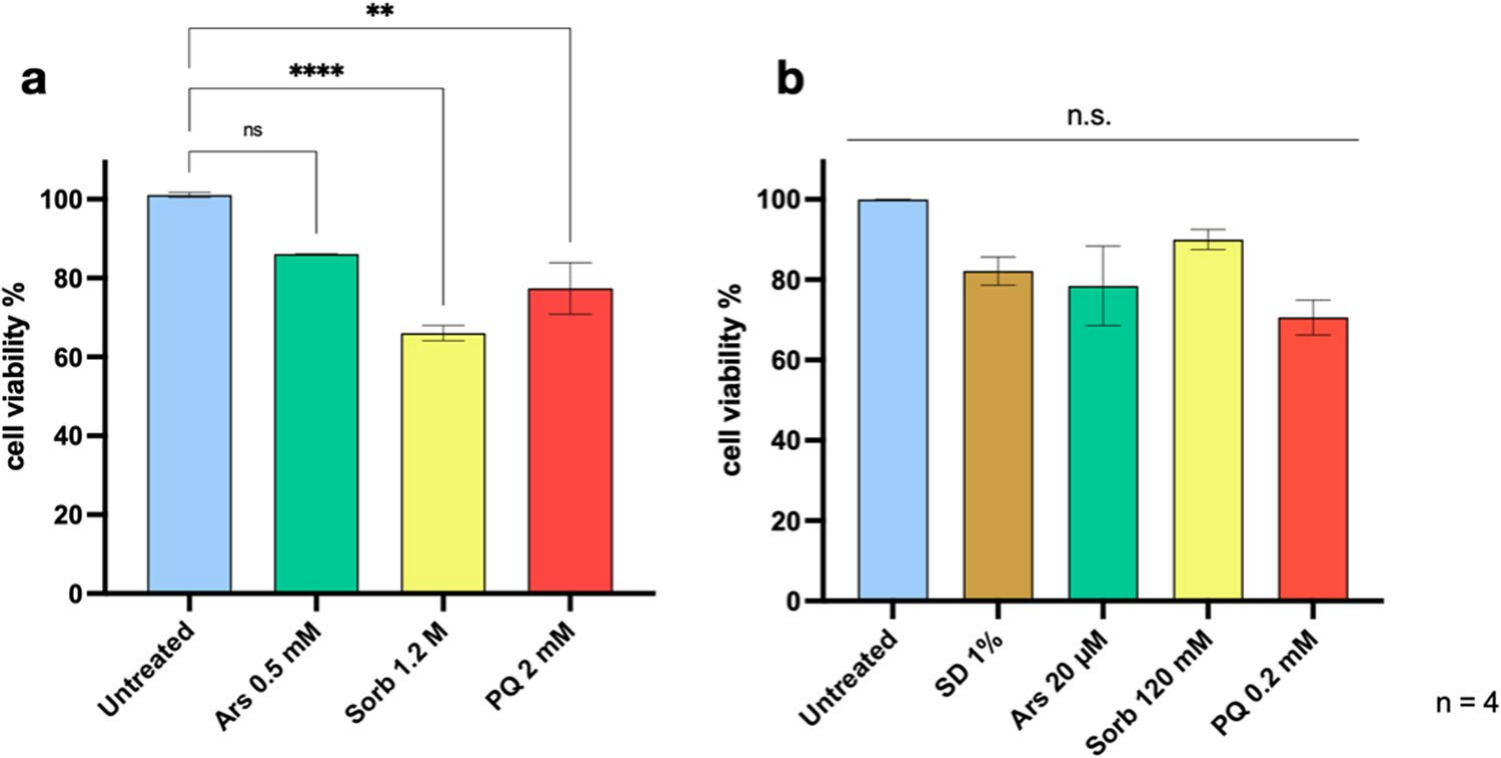
Assessment of cell viability after acute and chronic treatments. (**a**) Acute treatments causes a significant reduction in MTT absorbance, reflecting a reduced metabolic activity, upon treatment with 1.2 M Sorb (****, *p* < 0.0001) or 2 mM PQ (**, *p* < 0.01). Ars treatment failed to reach significance, although a trend towards reduced metabolic activity is observed compared to untreated cells. (**b**) Chronic treatment failed to reach significance in each condition tested, showing only a general trend in the reduction of cell viability as compared to untreated controls. Statistical analysis was performed on quadruplicates by One-way ANOVA with multiple comparisons against untreated control. (acute: F = 13.05; chronic: F = 1.851)

We first assessed the cytotoxicity of each stressor upon acute and chronic treatments through MTS absorbance. Acute Ars treatment resulted in a non-significant decrease in cell viability for each concentration tested compared to the untreated control. Likewise, the lowest tested concentration of Sorb and PQ (0.6 M and 1 mM, respectively) showed a non-significant reduction in MTS absorbance compared to the untreated control. On the other hand, higher concentrations of Sorb and PQ caused a significant reduction of cell viability (*n* = 4, *p* < 0.05 for Sorb 1 M; *p* < 0.001 for Sorb 1.2 M; *p* < 0.01 for PQ 2 mM; *p* < 0.001 for PQ 2.5 mM) (Suppl. Figure 1a). Working concentrations were selected as those providing the best balance between cell death and viability after acute stress (Fig. 1a). Following chronic treatments, a trend of decreasing cell viability could be observed, albeit not statistically significant (Suppl. Figure 1b). Thus, the highest concentration tested among all chronic conditions was considered as the working concentration for further experiments (Fig. 1b).

### RIPA-Insoluble Full-length TDP43 is Enriched upon Acute Stress

**Fig. 2:**
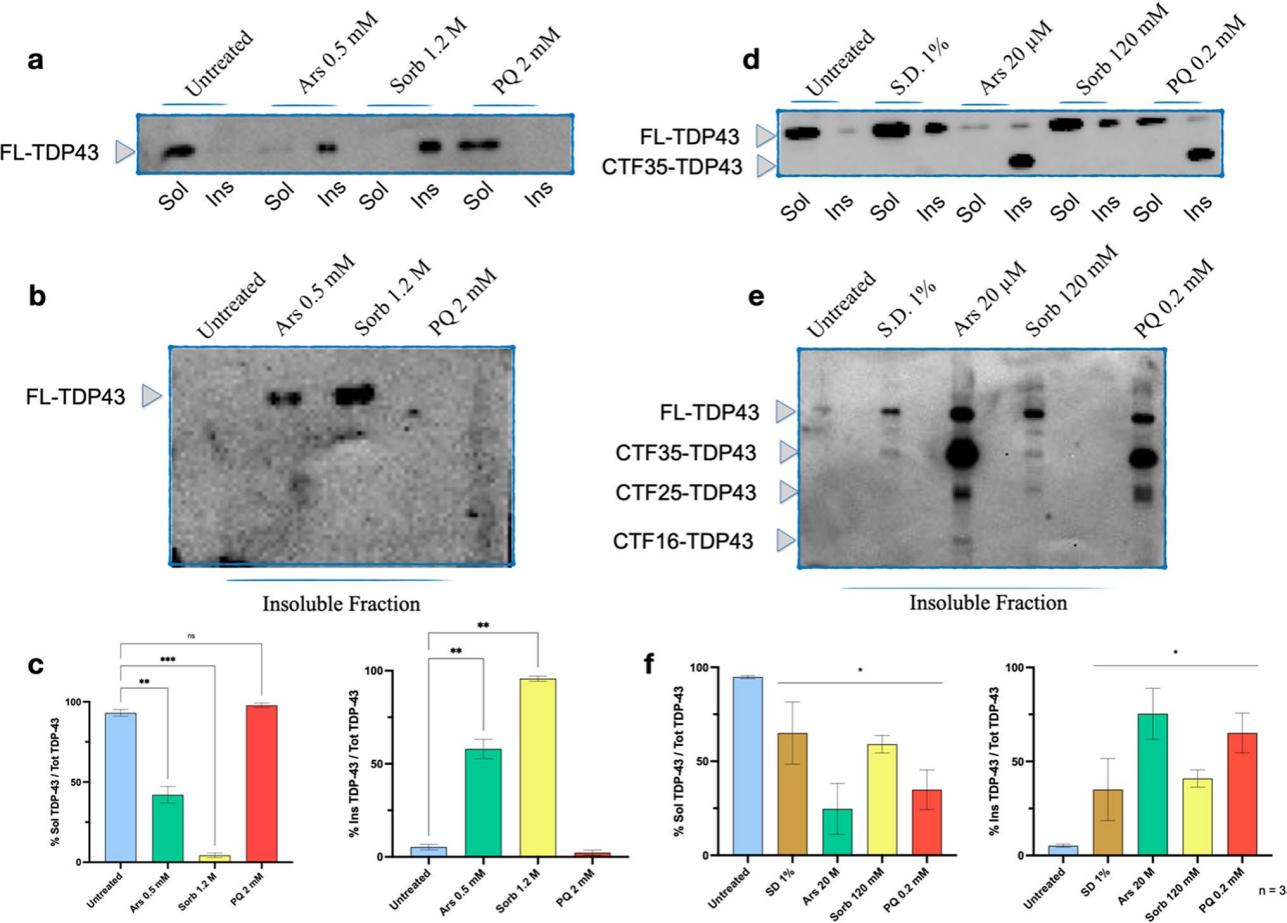
TDP43 solubility upon acute and chronic treatment. (**a**) Representative Western Blot showing the presence of RIPA-insoluble forms (Ins) of TDP43 after acute treatment with Ars and Sorb, while only RIPA-soluble (Sol) TDP43 could be found in untreated and acute PQ-treated samples. (**b**) Representative Western Blot showing a longer exposure performed on Ins fractions after acute stress. (**c**) Densitometric analysis was performed on biological triplicates as ratio between soluble or insoluble TDP43 and total TDP43. A significant reduction in the amount of Sol TDP43 was found between untreated samples and Ars-treated (*, *p* < 0.05) and between untreated and Sorb-treated samples (***, *p* < 0.001). Accordingly, a significant increase in the amount of Ins TDP43 was found between untreated samples and Ars-treated (**, *p* < 0.01). and between untreated and Sorb-treated samples (**, *p* < 0.01). (**d**) Representative Western Blot showing the presence of Ins TDP43 after chronic treatment in all stressful conditions tested. (**e**) Representative Western Blot showing a longer exposure of Ins fractions, revealing the presence of multiple TDP43 cleavage products. (**f**) Densitometric analysis was performed on triplicates as ratio between Sol or Ins TDP43 and total TDP43. A significant reduction in soluble TDP43 was found upon treatments compared to untreated control (*, *p* < 0.05). Each stressors elicited the formation of TDP43 insoluble species, with significant difference compared to untreated cells (*, *p* < 0.05). Data are shown as mean ± S.E.M. and were analyzed by One-way ANOVA with multiple comparisons using untreated control as reference (acute soluble: F = 152.1; acute insoluble: F = 154.8; chronic soluble: F = 6.101; chronic insoluble: F = 5.244)

We next analyzed the effect of acute stressors on TDP43 solubility. Cells were harvested at the end of each acute treatment. RIPA-soluble (Sol) and RIPA-insoluble (Ins) fractions were extracted, quantified by Bradford assay and separated by SDS-PAGE. Full length (FL)-TDP43 was detected in the Ins fractions of both Ars and Sorb-treated samples but neither in untreated nor in PQ-treated samples (Fig. 2a), indicating a shift in TDP43 solubility following Ars and Sorb treatment. To assess the eventual presence of TDP43 cleaved products, we tested a longer exposure of the Ins fractions only (Fig. 2b). The analysis under these detection conditions confirmed that FL-TDP43 was revealed only after treatment with both Ars and Sorb, and none of its cleavage products was virtually observed in either fraction. Densitometric analysis performed on biological triplicates normalized on GAPDH as an internal control showed that acute treatments did not cause a variation in the total amount of FL-TDP43, as expected [38] (Suppl. Figure 2a). Soluble FL-TDP43 was significantly reduced upon Ars and Sorb exposure (*n* = 3, *p* < 0.05 for Ars; *p* < 0.001 for Sorb). Accordingly, the amount of insoluble FL-TDP43 significantly increased after Ars (*n* = 3, *p* < 0.01) and Sorb (*n* = 3, *p* < 0.01) acute exposure (Fig. 2c). These results indicate that Ars and Sorb acute treatments converge on an increase of RIPA-insoluble FL-TDP43 without any TDP43 associated fragments, whilst acute PQ treatment appears to have no influence of TDP43 solubility in RIPA buffer. Interestingly, the amount of Ins FL-TDP43 seems to depend on the concentration of the stressor. Indeed, insoluble TDP43 species could not be detected with a low concentration of Sorb (0.6 M), are barely detectable at 0.8 M Sorb, and are well represented at increasing intensity at 1 M and 1.2 M (Suppl. Figure 2b).

### Pathology-associated TDP43 Fragments are Produced upon Chronic Stress

Similar to acute treatments, TDP43 solubility in RIPA buffer was assessed for chronically treated samples as well. Both soluble and insoluble FL-TDP43 bands were detected in each fraction upon chronic treatment, whilst only a small amount of FL-TDP43 could be observed in the Ins fraction of untreated samples (Fig. 2d). Ars- and PQ-treated samples showed a clear TDP43 positive band in the insoluble fraction with electrophoretic mobility of approximately 35 kDa, coherent with the production of the pathology-associated CTF-35 [13]. When only the Ins fractions were run and after longer exposure, various lower molecular weight TDP43 positive bands were detected (Fig. 2e). Specifically, 35 kDa band was detected in Ins fraction from SD 1%, Ars, Sorb and PQ treated samples, although with different intensity. Only Ars treatment produced a detectable band around 16 kDa, while multiple bands mapping at 25 kDa, coherent with the presence of the pathology-associated CTF-25 [13] were detected in Ars as well as in PQ-treated samples, while 25 kDa single band was detected in Sorb-treated samples. Remarkably, the TDP43 banding pattern following PQ incubation appears to be similar to Ars-treated samples, although no bands less than 25 kDa could be observed. Densitometric analysis was conducted after normalization against the GAPDH intensity signal deployed as internal control. As expected [38], the expression of FL-TDP43 did not appear to be regulated (Suppl. Figure 2c). A significant reduction of soluble TDP43 was found upon each treatment (*n* = 3, *p* < 0.05). Correspondingly, Ins TDP43, calculated as the sum of the intensity of every TDP43 band, was significantly increased in each treatment when compared to untreated control (Fig. 2f) (*n* = 3, *p* < 0.05).

These results suggest that fragments derived from endogenous TDP43 could be produced in SH-SY5Y cells upon chronic exposure to different forms of stressors, each resulting in a unique banding pattern.

### Acute Stress does not Cause an Increase in ThS Signal

**Fig. 3:**
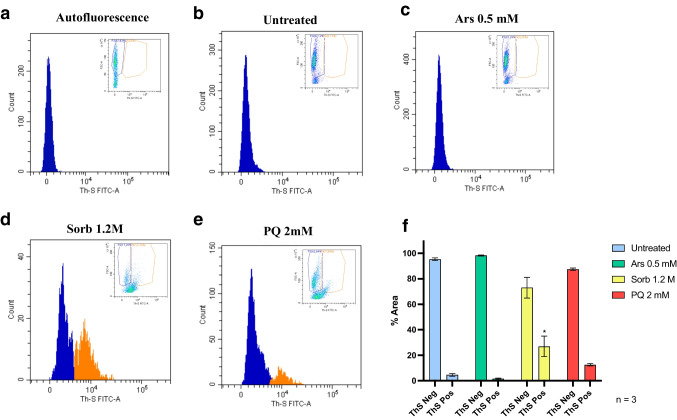
Overall amyloid burden after acute treatment detected by ThS staining through flow cytometry, plotting cell count versus ThS fluorescence emission. Blue curves represent ThS negative events, orange curves represent ThS positive events. (**a**) Autofluorescence and (**b**) untreated control showed no background staining; acute Ars (**c**) samples showed no increase of ThS signal. A mild but significant increase (* *p* < 0.05, *n* = 3) could be observed after acute Sorb treatment (**d**), whereas minimal but not significant increase in ThS signal could be observed upon PQ treatment (**e**). Statistical analysis was performed by One-way ANOVA with multiple comparisons against untreated control (**f**) (F = 5.960)

Next, we sought to gain insights into the nature of RIPA-insoluble TDP43 species produced by acute and chronic treatments. To this end, we set up a protocol for the detection of *bone fide* prion-like structures in living cells employing flow cytometry coupled with ThS amyloid-binding properties. Events read by flow cytometry were gated to exclude debris and doublet events as shown in Suppl. Figure 3, resulting in a final histogram plotting forward scatter versus ThS emission. Events selected by plotting forward scattering and ThS fluorescence are shown in the top-right box of each histogram plot in Fig. 3. Basal fluorescence of cells in absence of ThS (autofluorescence, Fig. 3a), as well as untreated control (Fig. 3b), are reported. Acute treatments failed to produce any ThS positive events, with only Sorb treatment producing a small increase in ThS emission (Fig. 3c-e). Statistical analysis of the ThS positive peak was conducted by One-way ANOVA with multiple comparisons against untreated control (Fig. 3f). Histograms report the percentage of ThS positive and ThS negative peaks (orange and blue, respectively) normalized against the sum of the two peaks (Fig. 3a). Statistical significance could be achieved only by Sorb treatment (Fig. 3d, *n* = 3, *p* < 0.05).

This result indicates that acute treatment with Ars and PQ does not lead to an increase in the total amount of ThS positive structures within living cells, whereas a small significant increase in ThS fluorescence could be detected upon acute Sorb exposure.

### Chronic Stress Causes an Overt Increase in ThS Signal

**Fig. 4:**
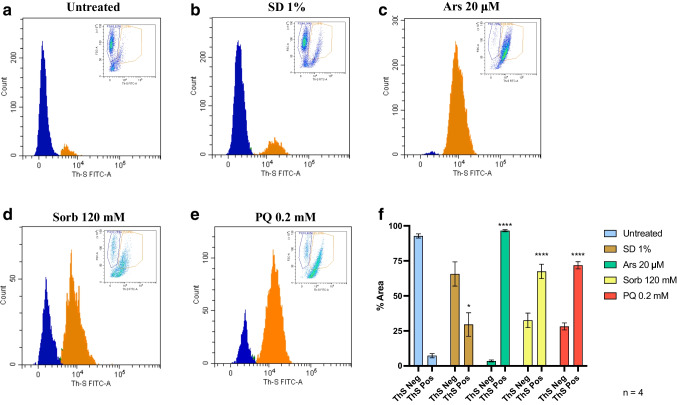
Overall amyloid burden after chronic treatment detected by ThS staining through flow cytometry, plotting cell count versus ThS fluorescence emission. Blue curves represent ThS negative events, orange curves represent ThS positive events. (**a**) No ThS fluorescence signal was observed in untreated cells. A mild but significant increase (* *p* < 0.05) in ThS signal could be detected upon chronic starvation (**b**), while Ars (**c**), Sorb (**d**) and PQ (**e**) chronic treatment caused an overt ThS positive peak (**** *p* < 0.0001, *n* = 4). Statistical analysis was performed by One-way ANOVA with multiple comparisons against untreated control (**f**) (F = 56.48)

The flow cytometry protocol was applied to chronically treated samples. The gating strategy for each chronic treatment is shown in Suppl. Figure 4. Autofluorescence sample reported no basal ThS positive staining (Fig. 4a), while untreated samples show minimal ThS emission (Fig. 4b). In sharp contrast to acute treated samples, chronic treatments all resulted in an evident increase of ThS fluorescence emission (Fig. 4b-e). SD 1% caused a mild but significant increase in ThS emission (Fig. 4b) Ars treatment caused an almost complete shift towards ThS positive events (Fig. 4c); Sorb and PQ showed ThS positive signals in more than half of the events (Fig. 4d, e). Statistical analysis of the ThS positive peak was conducted by One-way ANOVA with multiple comparisons against untreated control and histograms were produced as aforementioned (Fig. 4f). All chronic treatments showed a significant increase in ThS emission, with Ars treatment causing the highest increase of ThS signal (*n* = 3, *p* < 0.0001), followed by Sorb and PQ (*n* = 3, *p* < 0.0001) and SD (*n* = 3, *p* < 0.05) treatments.

These results highlight the ability of our flow cytometry protocol to stain in living cells *bona fide* amyloids with ThS, suggesting that these structures are formed by endogenous proteins upon prolonged stressful conditions.

### TDP43 is Mobilized into the Cytosol upon Acute Treatments but does not Co-localize with ThS Fluorescence

**Fig. 5:**
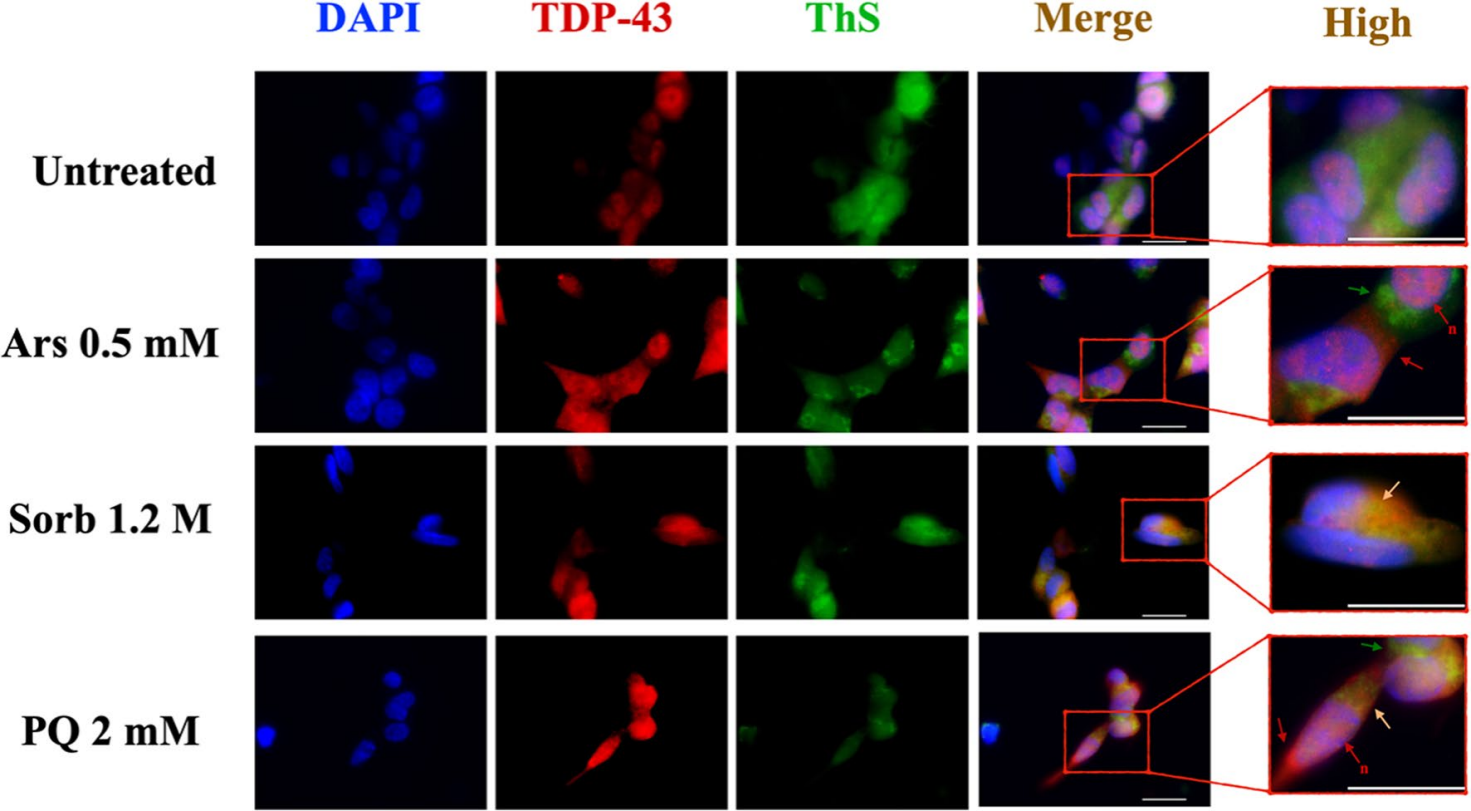
TDP43 mobilization upon acute treatments. Representative images of immunofluorescence staining of acute treated SH-SY5Y cells are shown for DAPI (blue), TDP43 (red) and ThS (green) and merged colors for each condition. Details are shown at greater magnification (labelled as “high”), in which arrows indicate ThS positive cytoplasmic puncta (green), TDP43 positive cytoplasmic puncta (red) and nuclear puncta (n, red) and ThS positive, TDP43 positive cytoplasmic puncta (yellow). Scale bar = 20 µm

TDP43 is known to be mobilized from the nucleus to the cytoplasm following stress induction [25–29]. We thus observed TDP43 aggregation by performing TDP43 immunofluorescence coupled with ThS staining. The presence of background signal from secondary antibody was excluded by staining untreated samples without primary antibody (Suppl. Figure 5a). untreated cells showed the staining with anti-TDP43 as mostly nuclear, in agreement with previous reports in SH-SY5Y cells [21, 29, 39]. Under acute stress conditions (Fig. 5), anti-TDP43 was detected in the cytosol in each condition tested, beside untreated controls (red arrows in magnified images labeled as “high”), appearing either as diffuse fluorescence (Sorb- and PQ-treated) or as bright puncta (Ars-treated). Moreover, intranuclear condensates of anti-TDP43 Ab could be spotted (red arrows, n). In untreated conditions, ThS fluorescence appeared as diffuse, staining the whole cell body. Upon treatment, clusters of ThS could be found throughout the cells (green arrows in magnified images). Notably, only acute Sorbitol treatment resulted in a clear co-localization between anti-TDP43 and ThS (orange arrows in magnified images). Acute Ars and PQ caused a partial mobilization of TDP43 outside the nucleus and minimal co-localization between ThS and anti-TDP43.

Overall, whereas each condition could cause part of TDP43 to be mobilized from the nucleus to the cytoplasm, only a little amount of TDP43 could be detected within cross-β positive structures observed by ThS fluorescence, suggesting that TDP43 may undertake different aggregation pathways under acute stress.

### TDP43 Shuttles into the Cytosol and Partly Co-localizes with ThS Fluorescence upon Chronic Treatment

**Fig. 6:**
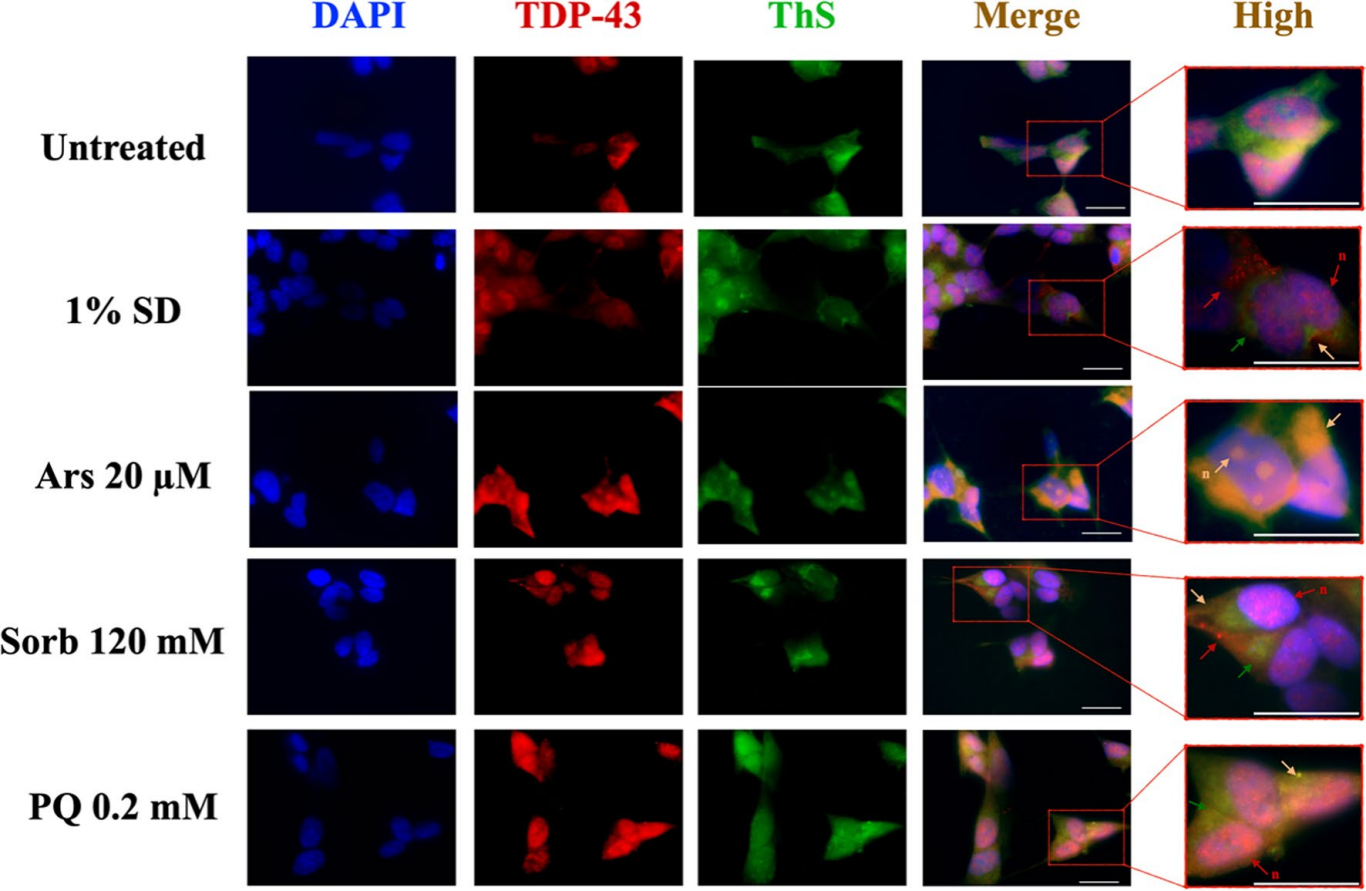
TDP43 mobilization upon chronic treatments. Representative images of IF staining of chronic treated SH-SY5Y cells are shown for DAPI (blue), TDP43 (red) and ThS (green) and merged colors for each condition. Details are shown at greater magnification (labelled as “high”), in which arrows indicate ThS positive cytoplasmic puncta (green), TDP43 positive cytoplasmic puncta (red) and nuclear puncta (n, red) and ThS positive, TDP43 positive cytoplasmic puncta (yellow). Scale bar = 20 µm

TDP43 mobilization and its association with cross-β structures under chronic stress were assessed using immunofluorescence staining coupled with ThS staining. The presence of background fluorescence from the secondary antibody was excluded by staining performed on untreated cells without primary antibody (Suppl. Figure 5b). untreated SH-SY5Y cells showed that TDP43 localizes mainly in the nucleus, whereas ThS staining resulted in weak and diffuse fluorescence emission, similar to what observed under acute condition. TDP43 + cytoplasmic puncta could be detected along each chronic treatment. ThS + /TDP43- spots could be detected, together with TDP43 + /ThS + aggregates, as shown by merged images (Fig. 6). Importantly, a fraction of TDP43 still retains its nuclear localization upon chronic treatment, forming intranuclear spots akin to those observed in acute conditions (red arrows, n). Punctiform, mostly ThS- / TDP43 + foci were found after SD 1%, although ThS + and TDP43 + aggregates could be observed as well. Such spots are characteristic of Ars-treated samples, where spots appear to be larger and less regular in shape. Here, nuclear aggregates positive to both TDP43 and ThS were also found. Sorb treatment resulted in the appearance of both round-shaped ThS-/TDP43 + spots, along with diffuse ThS staining and evidence of ThS + /TDP43 + structures. Lastly, PQ induced the formation of co-localized, extranuclear TDP43 + /ThS + spots, although nuclear TDP43 appears to be predominant.

Interestingly, co-localization between TDP43 and ThS staining was not uniform amongst treatments, with Ars showing the highest degree of co-localization, followed by PQ, Sorb and SD 1%.

### Phosphorylated TDP43 is Enriched in the Cytosolic Fraction upon Chronic Stress Induction

**Fig. 7:**
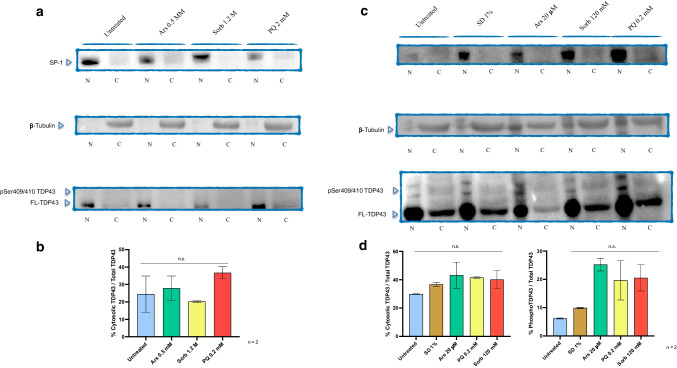
TDP43 subcellular localization after acute and chronic treatments. (**a**) Representative Western blots showing nuclear and cytosolic TDP43 upon acute stress. Nuclear and cytosolic markers (SP-1 and β-Tubulin, respectively) are shown as well. No phospho-TDP43 (pSer409/410) could be detected. (**b**) Densitometric analysis was performed on biological duplicates and expressed as ratio percentage between cytosolic TDP43 and total TDP43 calculated as sum of all TDP43 bands. No significant differences were found in this setting (One-way ANOVA, F = 2.225). (**c**) Representative Western blots showing nuclear and cytosolic TDP43 upon chronic stress along with nuclear and cytosolic markers. Cytosolic TDP43 was found in each condition tested, including untrated controls. Bands coherent with the presence of phosphoTDP43 could be found in every condition tested, with the highest signal deriving from PQ, Sorb and Ars. Although not significant, a trend in increased cytosolic and phosphorylated TDP43 could be detected compared to untreated controls (**d**) (One way ANOVA, F = 0.912 and F = 6.779, respectively. Data are shown as mean ± S.E.M

Finally, in order to validate the presence of cytosolic TDP43 observed by immunofluorescence, we separated the nuclear and cytosolic fractions after acute and chronic stress induction and probed them for total and phosphorylated TDP43 (Fig. 7). The purity of nuclear and cytosolic fractions was confirmed by specific markers for nuclear extract (SP-1) and cytosolic extract (β-tubulin). FL-TDP43 was found predominantly in the nucleus. As expected [21], approximately 25% of total FL-TDP43 could be found in the cytosolic fraction in untreated controls of both acute and chronic treatments. Upon acute treatment (Fig. 7a, Suppl. Figure 6a), neither phosphorylated forms of TDP43 nor an increase in cytosolic/total TDP43 ratio could be detected (Fig. 7b). An increased signal from cytosolic FL-TDP43 could be observed upon each chronic stressful condition compared to untreated control (Fig. 7c, Suppl. Figure 6b). SD1% caused the minimal increase in phospho-TDP43, whilst Ars, Sorb and PQ resulted in about 20% of phospho-TDP43 relative to total TDP43 (Fig. 7d). Lack of statistical significance could be due to the limited number of biological replicates (*n* = 2) and to the intrinsic high variability of a semi-quantitative approach such as the western blot. Nevertheless, a trend in the increase of cytosolic TDP43 and phospho-TDP43 could be found. Interestingly, when insoluble fractions derived from chronically treated samples were probed for phosphorylated TDP43, a clear band corresponding to phosphorylated FL-TDP43, as well as a band corresponding to phosphorylated CTF35, could be detected. This signal was strongest after Ars treatment and evident after PQ treatment, whereas Sorb and SD1% resulted in faint signal from the phosphorylated forms of TDP43 (Suppl. Figure 6c). Since this experiment was performed without phosphatase inhibitors, we speculate that this epitope is protected from endogenous phosphatases by being buried in insoluble aggregates. Although only preliminary, these results would suggest that upon prolonged stress TDP43 is mobilized in the cytosol, where it is phosphorylated at the pathogenic epitope S409/S410 and becomes insoluble in RIPA buffer.

## Discussion

Over the last two decades, the role of TDP43 misfolding and aggregation has been reported as central in the pathogenesis of ALS and FTLD [1–6]. TDP43 inclusions have also been associated with other neurodegenerative diseases, including Alzheimer’s, Parkinson’s and Huntington’s Diseases [40–43]. TDP43 pathological behaviour has been recapitulated both in animal and cellular-based experimental procedures, showing that TDP43 biology outside the nucleus could be central for its toxicity towards neurons.

Here we combined TDP43 biochemical analyses and immunofluorescence staining with the study of the overall amyloid burden through Thioflavin fluorescence in chronic, long-term stress paradigms and compared them to acute stress induction. For each treatment, results from cytotoxicity, solubility and flow cytometry assays, along with evidence obtained from immunostaining are summarized in Table 2. Our results may shed light on the importance of protein aggregation as a primarily physiological response toward different stress paradigms.

**Table 2:** Effect of Acute versus chronic stress. Summary of the results obtained by cytotoxicity. solubility. staining and flow cytometry assays in acute and chronic for each treatment

Stressor	Effect of Acute Stress	Effect of Chronic stress
Serum Deprivation (SD)	/	- Mild reduction in cell viability - Mild increase in total ThS - Formation of RIPA-insoluble FL-TDP43 and CTF35 - Cytosolic TDP43 ThS positive and ThS negative - Clusters of cytosolic ThS
Sodium Arsenite (Ars)	- Mild reduction in cell viability - No increase in total ThS - RIPA Insoluble FL-TDP43 - Cytosolic TDP43 ThS negative - Clusters of cytosolic ThS	- Non-significant reduction in cell viability - Overt increase in ThS - RIPA insoluble FL-TDP43, CTF35, CTF25 and CTF16 - Diffuse cytosolic TDP43 ThS positive
D-Sorbitol (Sorb)	- Significant reduction in cell viability - No increase in total ThS - RIPA Insoluble FL-TDP43 - Cytosolic TDP43 ThS positive	- Non-significant reduction in cell viability - Moderate increase in ThS - RIPA insoluble FL-TDP43, CTF35 and CT25 - Partial cytosolic co-localization between TDP43 and ThS
Paraquat (PQ)	- Significant reduction in cell viability - No increase in total ThS - Lack of RIPA insoluble TDP43 species - Minimal co-localization between TDP43 and ThS	- Non-significant reduction in cell viability - Overt increase in ThS - RIPA insoluble FL-TDP43, CTF35, CTF25 and CTF16 - Punctiform cytosolic TDP43 ThS positive

### Multiple Chronic Stress Paradigms Converge on the Formation of Pathology-associated TDP43 C-Terminal Fragments

Although the distribution and morphology of TDP43 species differ amongst subtypes of pathologies, the CTF-25 has been consistently identified as the major TDP43 product associated with neuronal inclusions found ion patients and is considered the main pathological signature of ALS and FTLD [3, 12–14]. However, cellular models aimed to recapitulate TDP-43 pathogenesis typically result in the production of the 35 kDa species (CTF-35), which is less represented in patients [14, 44–46]. The most common approach to stimulate TDP43 mobilization and fragmentation in cellular models consists in the administration of a stressor at high concentration for a short time. This acute approach, mostly conducted using Ars or Sorb as stressors, demonstrated the cytoplasmic association of TDP43 with stress granules, the increase in its insolubility and its liquid–liquid phase transition at varying degrees [22, 28–31, 47–52]. When reported, fragment formation is confined to CTF-35 and produced by either a combination of stress induction and/or genetic engineering to introduce aggregation-prone mutations in TDP43 [21, 23, 53, 54]. In our system, acute stress increased the insolubility of endogenous FL-TDP43 but, in line with previous reports, no fragments could be detected. Surprisingly, PQ failed to induce any shift in TDP43 solubility, suggesting that cytosolic TDP43 retains its solubility during this form of stress. The apparent disagreement with other studies reporting the increase in TDP43 insolubility upon acute PQ administration may be due to differences in protein extraction protocol or cell differentiation protocols [55, 56]. We aimed to develop a novel experimental design using a low concentration of stressors for an extended time, aiming to better recapitulate the biological processes that may take place during the silent phases of the pathology. Although chronic administration of stressors has been proposed, the time of administration varied between 6 and 30 h [30, 31], a timespan we deemed too short to elicit protein aggregation. Indeed, this process has been successfully achieved within hours only in seeded, cell-free systems such as the protein misfolding cyclic amplification or the real-time quaking-induced conversion technologies, where the kinetics of aggregation is dramatically enhanced by the presence of a misfolded pathogenic seed [57–59]. Thus, it is fathomable to speculate that an extended period may be required to produce protein aggregates in an in vivo setting. We hence extended the treatment up to 72 h and, by comparing the effect of acute versus chronic stress, we show that the long-term approach leads to the formation of both the commonly observed CTF-35 species and the hallmark CTF-25 TDP43 products derived from the endogenous TDP43 protein. Multiple bands could be observed in RIPA insoluble fractions of chronically treated samples, spanning from 35 to 16 kDa. Importantly, TDP43 fragments appear to be produced regardless of the source of the stress, as both oxidative (Ars, PQ), osmotic (Sorb) and environmental (SD 1%) stress converge in the appearance of insoluble, fragmented species of TDP43. Moreover, the banding pattern differs both in intensity and composition, suggesting that different cleavages may be involved in each stress response. Interestingly, phosphorylated, insoluble FL-TDP43 and CTF35 could be detected even in the absence of phosphatase inhibitors, suggesting that they might be protected from endogenous phosphatases due to their increased insolubility, which could indicate that these residues are buried within protein aggregates.

More studies are needed to elucidate the events during this process, such as which protease is involved and whether post-translational modifications are relevant in each stress paradigm. Nonetheless, here we report for the first time to our knowledge the production of endogenous TDP43 pathology-associated fragments in a physiological context, without the need for introducing an exogenous protein or a pathology-associated mutation.

### The Overall Amyloid Burden Increases in Response to Chronic Treatment

Since its discovery as the main protein found in inclusions in post-mortem brains of patients affected by ALS [1–4], multiple reports highlighted the ability of TDP43 to follow different routes of aggregation. Here, we sought to investigate TDP43 solid-phase aggregation pathway by exploiting the ability of thioflavin dyes to bind to cross-β structures, the building blocks for prion-like aggregation [52–62]. Liquid–liquid phase separation of TDP43, as well as its association with stress granules, appear to rely on alpha-helical structural features [63, 64]. Therefore, the discrimination of the aggregation pathway could be achieved by ThS binding, as this dye recognizes solid-phase aggregation but does not stain stress granules nor other liquid-demixed organelles.

By combining microscope images acquired after ThS staining with flow cytometry, we identified both ThS fluorescence relative to TDP43 localization and the overall amount of ThS binding molecules after long-term stress induction. Although thioflavin-coupled flow cytometry has been already reported as a suitable methodology to detect the overall amyloid burden in living cells, it has been mostly applied either to cell-free settings or to systems overexpressing the aggregating proteins in non-mammalian cells such as bacteria and yeasts [65–69]. Here we reported the formation of *bona fide* protein aggregates in human cells triggered by chronic stress induction. The mild increase in ThS fluorescence observed under acute Sorb treatment could be due to cell shrinkage after hyperosmotic stress. The reduction of cell volume causes a general increase in protein concentration, which is known to accelerate protein aggregation [70–72]. Fluorescence imaging confirmed that, upon chronic stress, ThS fluorescence co-localizes with cytosolic TDP43.

Subcellular fractionation further suggested that chronic stress causes the mobilization of TDP43 from the nucleus to the cytoplasm, where it becomes phosphorylated at the pathogenic residue Ser409/410.

Interestingly, the overt increase in ThS observed by flow cytometry is less apparent after immunofluorescence staining. This discrepancy may be attributed to the handling of samples: for flow cytometry, cells are collected by centrifugation, implying that detached cells are also collected with adherent ones; on the other hand, immunofluorescence stains only adherent cells, as cells in suspension are washed away, although they are still vital according to MTS assays.

Nevertheless, the results appear to be in good agreement when comparing each treatment. ThS intensity was lowest both by flow cytometry and immunostaining in SD 1% treated samples, whilst the highest signal in both methodologies was derived from Ars-treated samples. Notably, the same trend could be observed in the intensity of TDP43 fragments, although our study lacks direct evidence of their eventual contribution to the increase in ThS fluorescence observed by flow cytometry.

More studies are required to dissect the contribution of ThS-positive structures during the stress response. However, flow cytometry appears to be a suitable methodology for the detection of protein aggregates and could be of great importance for both basic research and future diagnostic purposes.

### Functional Amyloids: are Protein Aggregates made up of Pathology-related Proteins Inherently Toxic?

Our results point toward the formation of cross-β enriched structures as a physiological cellular response against chronic stress, with TDP43 localizing with these cytosolic structures. In parallel, biochemical evidence suggests that TDP43 is cleaved in multiple C-terminal fragments. Although the process of protein aggregation along the prion-like pathway is considered the main pathogenetic mechanism behind most neurodegenerative diseases, several studies reported the existence of functional amyloids playing physiological roles in virtually every kingdom of life, spanning from bacteria to mammals and including yeasts and plants [73–78]. However, few studies suggested a putative physiological function of amyloids associated with proteins related to neurodegeneration. The prion protein, for instance, was shown to aggregate over weeks following morphine withdrawal in rats [79]. Similarly, Tau protein was found to form neurofibrillary-like structures in hibernating mammals [80, 81]. Both experimental systems rely on chronic stress induction in animal models, akin to what we performed using chronic stress in a cellular model. We propose that TDP43 may exert a similar role, with protein aggregation functioning as a putative response mechanism against long-term insults. It will be interesting to evaluate whether ThS positive structures, as well as TDP43 fragments, are rescued upon the termination of the stress, as it would indicate the presence of a physiological disaggregating system within cells that could be of utmost importance for the development of therapeutic strategies against protein aggregation. On the other hand, should the stress be maintained, the aggregation process may become irreversible, leading to neuronal death and the spreading of the pathology.

Our study lacks an in-depth characterization of TDP43 in terms of post-translational modifications, proteases involved, type of oligomers and other pathways involved such as apoptosis and autophagy, as neurodegenerative-related proteins have been widely reported to be associated with these cellular functions [82–86]. Several pathways have been implicated in the production of pathogenic TDP43-CTFs. Mutagenesis experiments indicated that TDP43 is firstly cleaved after Asp174 by Caspase4, producing CTF25. This fragment activates downstream Caspase3/7, producing CTF35 [87]. Studies conducted in mice and non-human primates’ models of ALS further confirmed that CTF25 is produced upon Caspase4 cleavage [88]. Caspase-independent pathways have also been reported for the formation of pathogenic TDP43-CTFs. Ca^2+^-dependent cysteine protease Calpains were reported to generate in vitro C-terminal TDP43 fragments spanning from 33 to 36 kDa, independent from Caspases activation. These fragments appear to be downstream of the activation of the RNA editing enzyme ADAR2 and formed after cleavage by Calpains at multiple residues spanning from residues 229 and 346 of TDP43 [89]. Due to the heterogeneity of fragments found in our experimental setup, it is fathomable to speculate that multiple pathways could be involved in TDP43-CTFs generation upon chronic treatment. More studies will be needed to elucidate the precise molecular mechanism(s) involved in TDP43 fragmentation during various prolonged stress.

Besides TDP43, recent evidence highlighted the importance of other aggregating-prone proteins, such as TMEM106B, in the onset of ALS [90, 91]. More studies are clearly required to elucidate the contribution of protein aggregation in the early stages of neurodegenerative diseases, including but not limited to TDP43. Our study aimed to highlight the importance of this mechanism as a putative endogenous response that cells deploy to face chronic stressful conditions. We speculate that the aggregation of proteins related to neurodegenerative diseases may first serve a physiological role and only in time become a pathogenic mechanism, but more effort in this direction is needed to understand this ambiguous aspect of cell biology.

### Supplementary Information

Below is the link to the electronic supplementary material.

Supplementary file1 (PDF 66667 KB)

## Data Availability

The authors confirm that the data supporting the findings of this study are available within the article [and/or] its supplementary materials.
