# Supplementary material for: Different Chronic Stress Paradigms Converge on Endogenous TDP43 Cleavage and Aggregation
Source: Mol Neurobiol. 2023 Jul 14;60(11):6346–61. doi: 10.1007/s12035-023-03455-z (PMC10533643; doi:10.1007/s12035-023-03455-z)
Supplement: Supplementary file 1 — Supplementary file1 (PDF 66667 KB) [file 12035_2023_3455_MOESM1_ESM.pdf]

Suppl. Figure 1. Cytotoxicity of growing concentrations of acute and chronic stressors

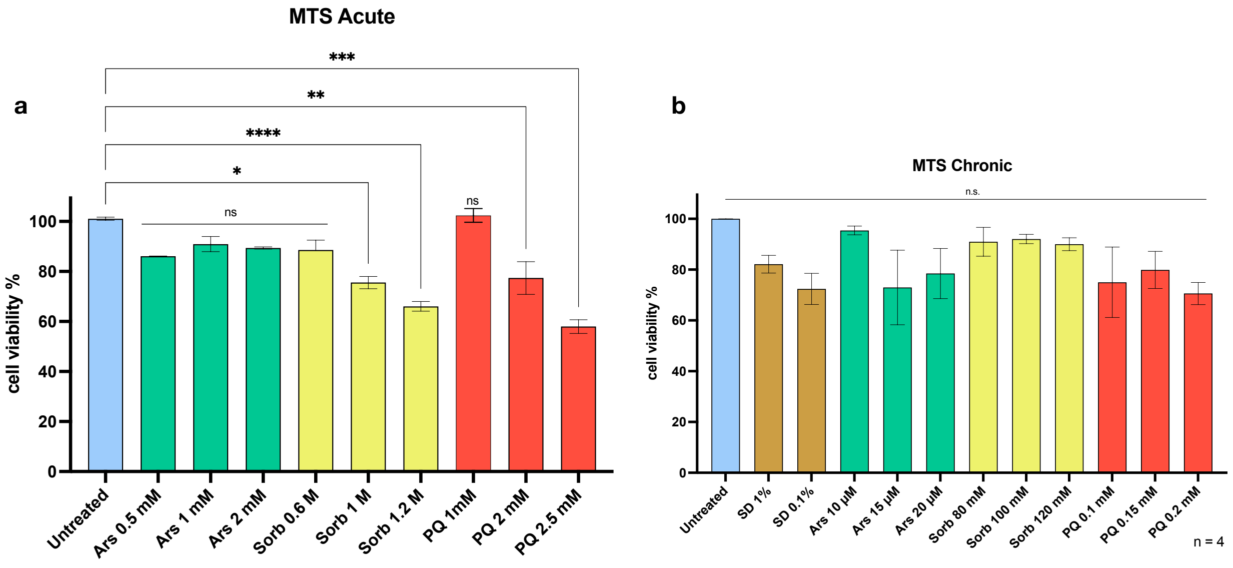

Supplemental Figure 1. Assessment of cell viability after growing concentrations of acute and chronic treatments. (a) Acute treatments caused a significant reduction in MTS absorbance upon treatment with 1 M and 1.2 M Sorb, and 2 mM and 2.5 mM PQ. Ars treatment failed to reach significance at every concentrations tested, with only a trend towards reduced metabolic activity compared to untreated cells. (b) Chronic treatment failed to reach significance in each condition tested, showing only a trend in the reduction of cell viability as compared to untreated controls. Statistical analysis was performed by One-way ANOVA with multiple comparisons against untreated control. ** $p<0.01$; *** $p<0.001$. Acute: $F = 13.05$; Chronic: $F = 1.851$ From this panel of concentrations. working concentrations for each stressor were selected.

Suppl. Figure 2. Uncropped Blots and normalization

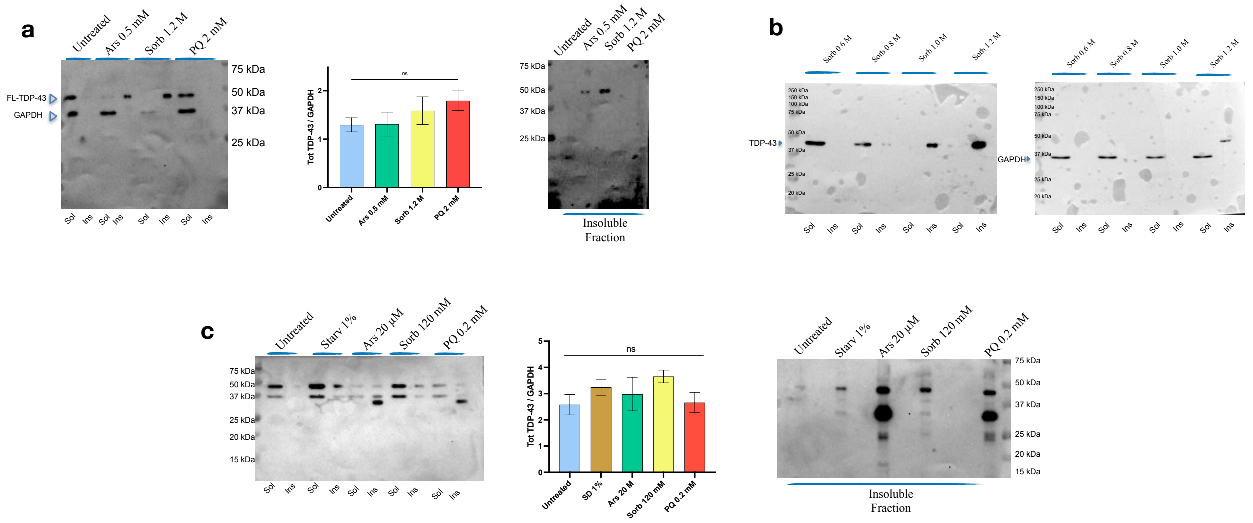

Supplemental Figure 2. (a) Uncropped Western Blot showing TDP43 bands in RIPA-Soluble and RIPA-Insoluble fractions following acute treatments with GAPDH as internal control. Densitometric analysis was performed on triplicates on the ratio between total TDP43 (as sum of each TDP43 band) and GAPDH, showing non-significant differences among treatments in the amount of total TDP43. Data are shown as mean +/- S.E.M. and were analyzed by One-way ANOVA with multiple comparisons using untreated control as reference. Uncropped Western Blot on a 10% Acrylamide gel with TDP43 bands in RIPA-Insoluble fractions with longer exposure is shown as well. (b) Uncropped Western Blot showing TDP43 bands in RIPA-Soluble and RIPA-Insoluble fractions following acute treatments with increasing concentrations of Sorbitol with GAPDH used as internal control. (c) Uncropped Western Blot on a 12.5% Acrylamide gel showing TDP43 bands in RIPA-Soluble and RIPA-Insoluble fractions following chronic treatment, with GAPDH used as internal control. CTF-35 is visible at low exposure in the insoluble fraction of Ars and PQ treated samples, and uncropped Western Blot on a 10% Acrylamide gel shows TDP43 bands in RIPA-Insoluble fractions after longer exposure. No significant difference was found among treatments in the amount of total TDP43, calculated as ratio between total TDP43 (as sum of each TDP43 bands) and GAPDH. Data are shown as mean +/- S.E.M. and were analyzed by One-way ANOVA with multiple comparisons using untreated control as reference. (acute soluble: $F = 152.1$; acute insoluble: $F = 154.8$; chronic soluble: $F = 6.101$; chronic insoluble: $F = 5.244$).

Suppl. Figure 3. Gating strategy for flow cytometry upon acute treatment

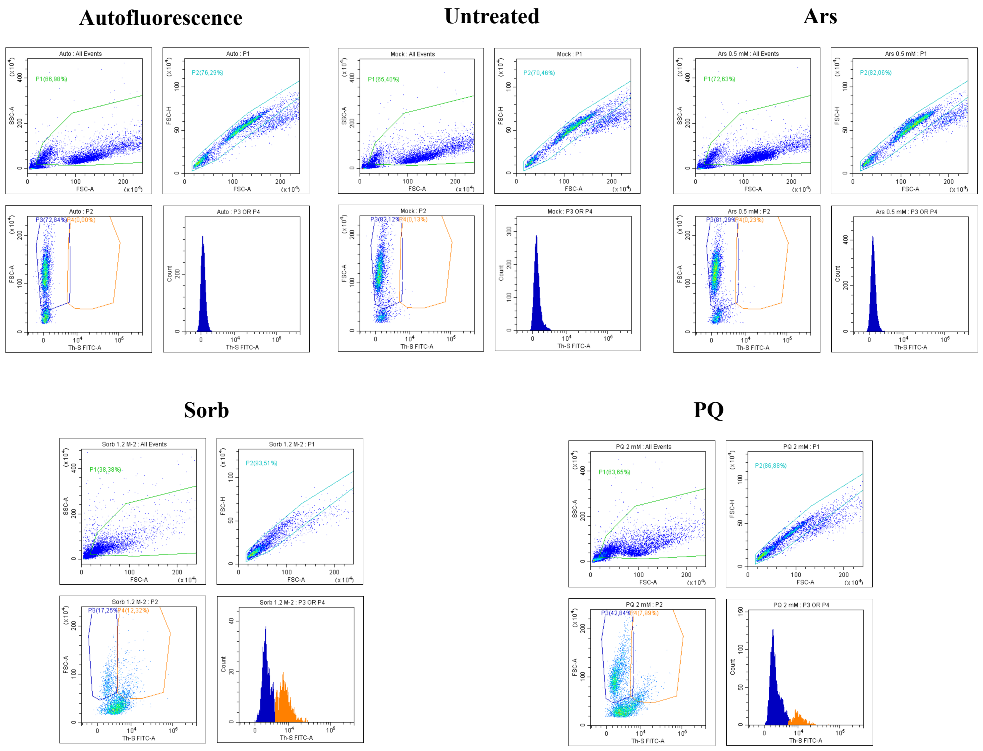

Supplemental Figure 3. Gating strategy for Flow cytometry for Thioflavin-S staining after acute stress. Cells were first gated (P1) by Forward Scattering (FSC) and Side Scattering (SSC) signals (upper left) to exclude debris; height versus area of FSC dot plot was used to exclude doublet events and cell clumps (P2, upper right); Cells in P2 were gated again (P3 and P4) by FSC-A and Th-S emission fluorescence, measured on a FITC detector (lower left). Th-S fluorescence intensity of both P3 and P4 cells vs count was represented in histogram plots (lower right).

Suppl. Figure 4. Gating strategy for flow cytometry upon chronic treatment

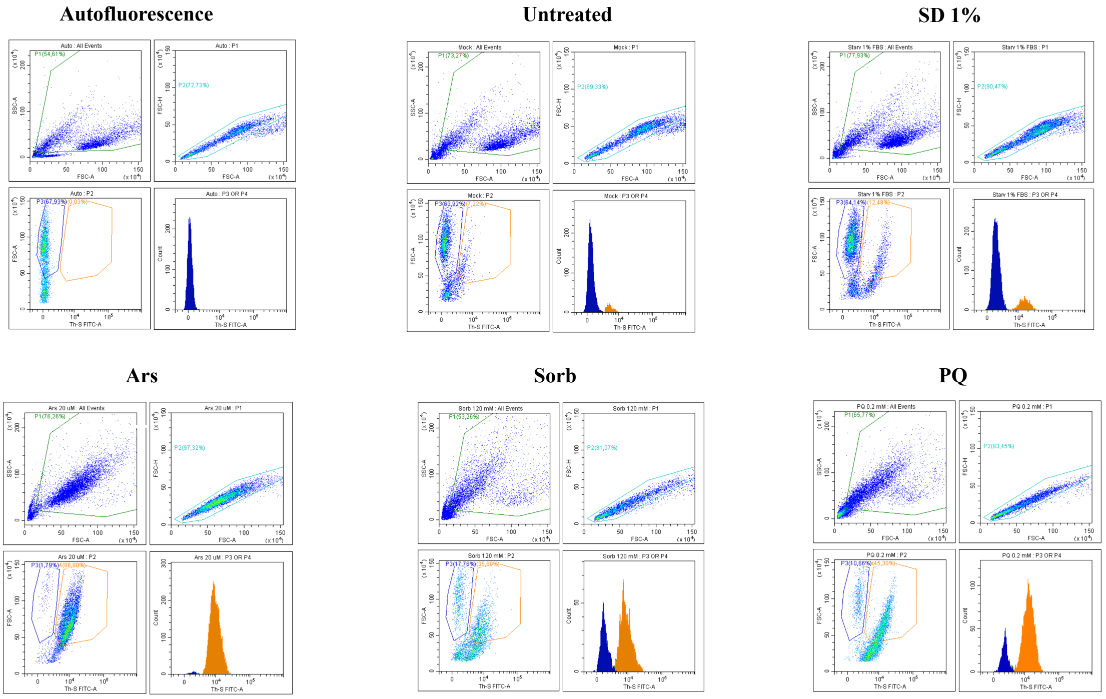

Supplemental Figure 4. Gating strategy for Flow cytometry for Thioflavin-S staining after chronic stress. Cells were first gated (P1) by Forward Scattering (FSC) and Side Scattering (SSC) signals (upper left) to exclude debris; height versus area of FSC dot plot was used to exclude doublet events asnd cell clumps (P2, upper right); Cells in P2 were gated again (P3 and P4) by FSC-A and Th-S emission fluorescence, measured on a FITC detector (lower left). Th-S fluorescence intensity of both P3 and P4 cells vs count was represented in histogram plots (lower right).

Suppl. Figure 5. Background fluorescence of ThS and secondary antibody after acute and chronic stress

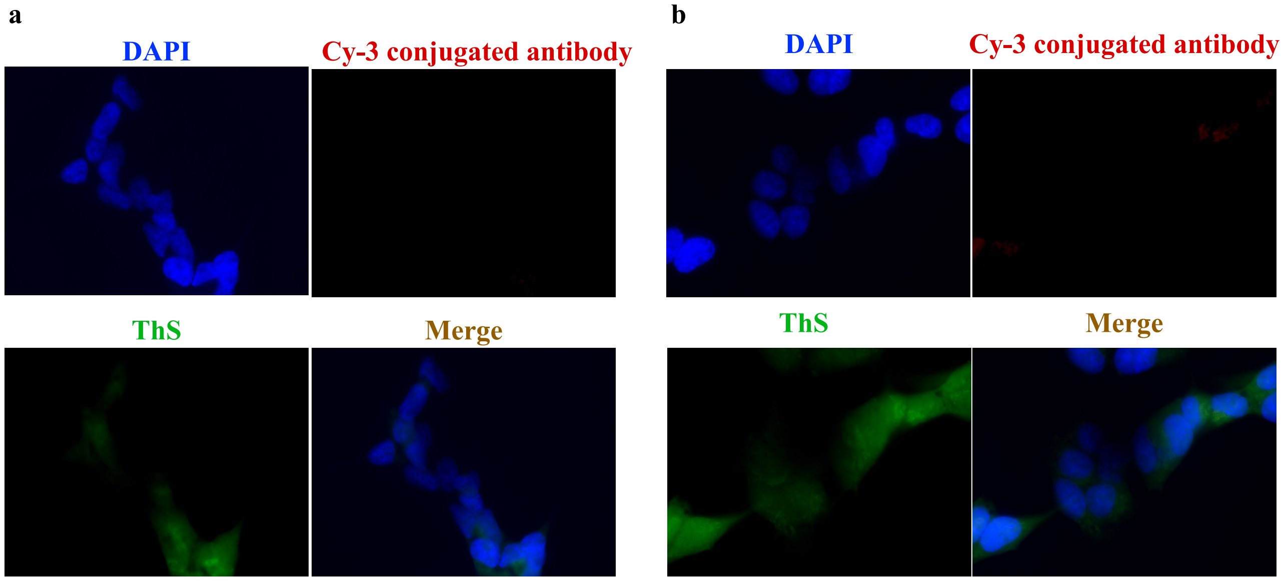

Supplemental Figure 5. Background fluorescence during IF experiments. (a) Immunofluorescence staining was performed in untreated treated SH-SY5Y cells using only secondary antibody, ThS and DAPI. No background fluorescence in the red channel was observed, indicating that the secondary antibody does not stain non-TDP-43 molecules. ThS and DAPI show also no background fluorescence, as DAPI stained only nuclear structures while ThS resulted in faint diffuse fluorescence. (b) Background fluorescence following chronic treatment. Immunofluorescence staining was performed in untreated SH-SY5Y cells using only secondary antibody, ThS and DAPI. Similar to acute-treated samples, no background fluorescence in the red channel was observed, indicating that the secondary antibody does not stain non-TDP-43 molecules. ThS and DAPI show also no background fluorescence. as DAPI stained only nuclear structures while ThS resulted in faint diffuse fluorescence.

Suppl. Figure 6. TDP43 nuclear and cytoplasmic localization

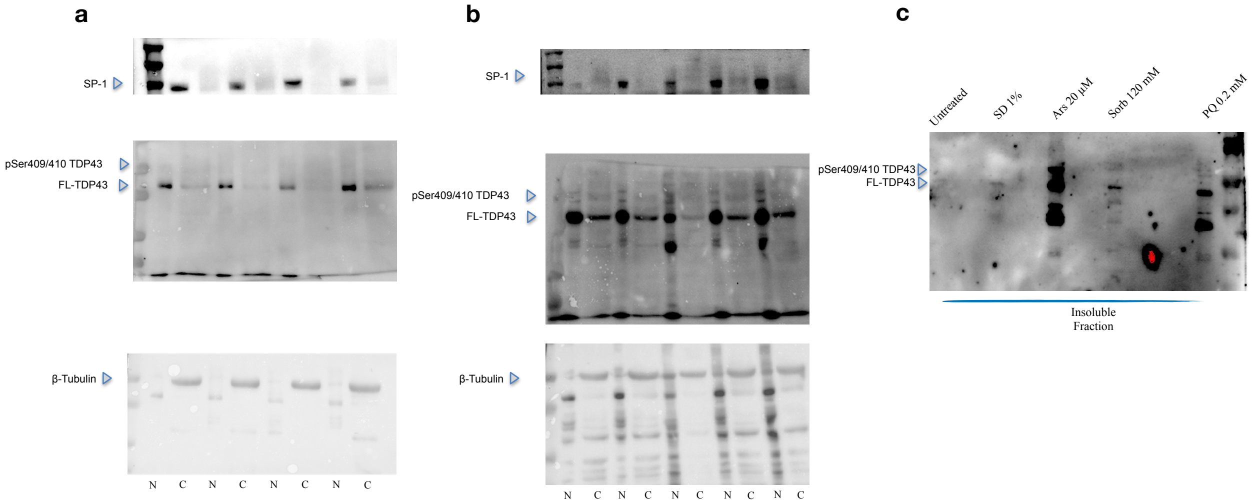

Supplemental Figure 6. (a) Uncropped Western Blot showing nuclear and cytosolic TDP43 and phospho-TDP43 (pSer409/410 TDP43) after acute stress, together with cytosolic and nuclear markers (β-Tubulin and SP-1, respectively). (b) Uncropped Western Blot showing nuclear and cytosolic TDP43 and pSer409/410 TDP43 after chronic stress, together with cytosolic and nuclear markers. (c) Uncropped Western Blot on a 10% Acrylamide gel shows TDP43 and pSer409/410 TDP43 bands in RIPA-Insoluble fractions after longer exposure.
